# Rifampicin Resistant Tuberculosis Among Presumptive Pulmonary Tuberculosis in Province Referral Hospital, Indonesia: Dynamic Cases of a 7-Year Report

**DOI:** 10.34172/jrhs.2024.136

**Published:** 2024-03-15

**Authors:** Novi Maulina, Zinatul Hayati, Kartini Hasballah, Zulkarnain Zulkarnain, Ika Waraztuty, Azzaura Defadheandra

**Affiliations:** ^1^Microbiology Department, Faculty of Medicine, Universitas Syiah Kuala, Banda Aceh, 23116, Indonesia; ^2^Pharmacology Department, Faculty of Medicine, Universitas Syiah Kuala, Banda Aceh, 23116, Indonesia; ^3^Physiology Department, Faculty of Medicine, Universitas Syiah Kuala, Banda Aceh, 23116, Indonesia; ^4^Anatomy Department, Faculty of Medicine, Universitas Syiah Kuala, Banda Aceh, 23116, Indonesia

**Keywords:** *Mycobacterium tuberculosis*, Indonesia, Sociodemographic factors

## Abstract

**Background:** Indonesia has the second highest tuberculosis (TB) cases globally. This study aimed to determine the sociodemographic factors associated with TB and rifampicin-resistant tuberculosis (RR-TB) cases among presumptive pulmonary TB patients in Aceh Referral Hospital.

**Study Design:** A retrospective cross-sectional study.

**Methods:** A retrospective cross-sectional review of presumptive pulmonary TB patients having a sputum test at the clinical microbiology laboratory was conducted from January 2015 to December 2021. Patient characteristics and drug susceptibility data were abstracted from the hospital information system of TB (SITB) and analyzed by univariate and bivariate analysis.

**Results:** The *Mycobacterium tuberculosis* (MTB) was detected in 32.8% sample (1,521/4,637). Of the TB-confirmed cases, 14.1% (215/1,521) were resistant to rifampicin (RR-TB). Most of them were male patients (71.63%), were in the age range of 35–54 years (48.7%), lived in rural areas of the country (56.3%), and were previously TB-treated cases (65.5%). Overall, 35–44-year-old patients (adjusted odds ratio [AOR]=2.11, 95% CI=1.25, 3.5, *P*<0.05) were more likely to have RR-TB compared to>65-year-old patients. Gender and residence were not associated with RR-TB (*P*>0.05). Case detection decreased in pandemic conditions (19.5% in 2019 to 13.9% and 7.91% in 2020 and 2021, respectively).

**Conclusion:** The findings revealed the dynamic cases and sociodemographic factors of TB and RR-TB in a province referral hospital in Indonesia for 7 years. The cases of TB and RR-TB among presumptive TB patients were 32.8% and 14.1%, respectively. The cases were found to be more noticeable in males, adults (45–54 years old), and patients residing in rural areas.

## Background

 Tuberculosis (TB) is an ancient infectious disease caused by acid-fast bacilli and is still a global public health concern.^1,2^ In 2021, the World Health Organization (WHO) reported that 10.6 million people were infected with *Mycobacteria tuberculosis* (MTB), and 1.6 million deaths occurred globally due to the disease. The establishment and transmission of multidrug-resistant TB (MDR-TB) is a crucial obstacle to controlling TB globally since this disease cannot be treated with currently used TB drugs.^3^ Second-line drugs that are time-consuming and costly are used to treat MDR cases.^4^

 Indonesia is the second leading country for TB cases globally.^3^ In 2022, there were be 724 309 new TB cases, with 12 531 cases of RR/MDR-TB. The number has potentially increased, with only 68% of the detected cases receiving treatment.^3,5^ As one of the provinces in Indonesia, Aceh contributed to a high number of TB cases. In 2022, 75% of drug-resistant TB cases were enrolled, and the treatment success rate (TSR) was 51%. As the government target for TSR is 80%, it is still necessary to make extra efforts to find the case earlier and optimize patient treatment.^5^ Located in the westernmost province of Sumatra Island, Indonesia, Dr. Zainoel Abidin Hospital serves as a prior referral hospital for 23 regions in the capital city of Aceh.^6^ The hospital has followed a national TB program using GeneXpert since mid-2014 to detect both MTB and the RR gene with high sensitivity.^7,8^

 Rifampicin resistance is a proxy marker for more than 90% of MDR-TB cases.^9^ Rifampicin was an important first-line TB drug and has been used for more than half a century.^10^ The bactericidal effect of rifampicin was achieved by its ability to bind with the RNA polymerase of MTB and interfere with its protein synthesis.^11^ Rifampicin resistance-related mutations are mostly located in the rifampin resistance-determining region of the RNA polymerase β subunit (*rpo β *gene),^12^ which makes genotypic-based drug-susceptibility testing (DST) of rifampicin more advantageous over other drugs. Repeated TB medication in relapse or loss-to-follow-up patients with cycles of MTB killing during early TB treatment and re-growth after treatment contributes to RR-TB. The GeneXpert MTB/RIF (Cepheid Inc., USA)^13^ and Genotype MTBDRplus (Hain Lifescience Inc., Germany)^14^ have been endorsed by the WHO as rapid genotypic rifampicin susceptibility tests.^15,16^

 GeneXpert MTB/RIF assay is a programmed, cartridge-based real-time polymerase chain reaction to detect MTB and the rifampicin resistance gene using molecular beacons within two hours.^17,18^ It was helpful in the rapid diagnosis of RR/MDR-TB among presumptive TB cases. A high prevalence rate of drug-resistant TB was reported from national data; however, there is a paucity of RR-TB magnitude and its associated factors in the study area. Therefore, this study sought to determine the TB and RR-TB cases and associated factors among presumptive pulmonary TB patients in the province referral hospital from 2015 to 2021.

## Methods

###  Study area

 This study collected retrospective data from presumptive TB patients receiving care at Aceh Province Referral Hospital (Dr. Zainoel Abidin Hospital) between January 2015 and December 2021. Aceh was the westernmost province of Indonesia, located on the northern end of Sumatra Island. The city currently has a population of 5 529 773 residents in 18 regencies and 5 cities, with Banda Aceh as the capital city of this special territory. The province has 24 referral hospitals and 98 health facilities for drug-resistant TB. Located in the capital city of Aceh, Dr. Zainoel Abidin Hospital serves as the main referral province hospital for 23 regencies and cities in the province.^5,6^

###  Study population

**Figure 1:**
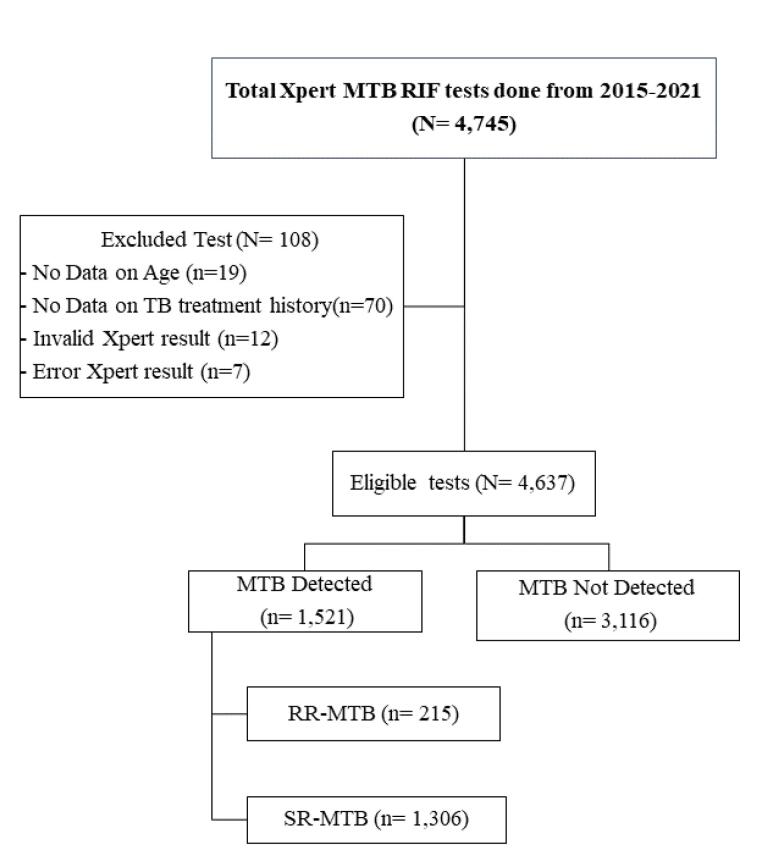
Flowchart for Inclusion and Exclusion Criteria

 Presumptive TB patients at province referral hospitals constituted the study population. The inclusion criterion was the involvement of all adult patients ( ≥ 17 years old), both male and female patients with DST results from the GeneXpert platform, while cases without DST results and uncompleted data were excluded from the study (Figure 1).

 New presumptive TB cases were subjected to DST using GeneXpert to detect both MTB and resistance to the drug. The test was performed using version 4 cartridges according to the kit instructions. The reagent contained NaOH and isopropyl alcohol in a 2:1 ratio to the collected sputum in a 15mL sterile container from each patient to kill the MTB and liquefy the samples. Then, it was manually shaken vigorously and incubated for 10 minutes before being re-shaken and re-incubated for another 10 minutes at room temperature. A sterile disposable pipette was used to transfer 2 mL of the material to the test cartridge (equipped with kits). Cartridges were put into the GeneXpert machine, and the software provided data interpretation from MTB/RIF tests after 90 minutes.^17^

 Patient sociodemographic characteristics (age, gender, patient residence [urban/peri-urban, rural, and others], patient category [new cases, relapse cases, loss-to-follow-up, and failure cases], and laboratory data [sputum smear and GeneXpert test results]) were extracted from the National TB Program electronic TB register (Information System of TB/SITB). A specific checklist form was utilized to abstract data from the reviewed laboratory results.

###  Analysis of samples by GeneXpert MTB/RIF assay

 The resistance of rifampicin was interpreted as the percentage of colonies that grew at the critical concentrations of the drug (40 µg/mL for Rif). The usual resistance criteria (i.e., 1% for all drugs) were used for the analysis. The invalid, error, or no result would be rerun if there was available material.^18^

###  Data analysis

 The data were abstracted in Excel and exported to Statistical Package for Social Sciences (SPSS) software (version 21, SPSS, Inc., Chicago, Illinois, USA) for analysis. The RR-TB cases among patients and each year of study were computed, and the association between RR-TB and sociodemographic factors was determined by bivariate analysis. The results were noted as crude odds ratios and adjusted odds ratios with a 95% confidence interval (CI). Significance for all statistical analyses was noted if the *P* value was smaller than 0.05.

## Results

 The records of 4745 presumptive TB patients who had DST using GeneXpert from 2015 to 2021 in Aceh Referral Hospital were assessed, and 4,637 eligible cases were included and abstracted. The positivity of MTB among presumptive TB patients was 32.8% (1521/4637). Of all MTB confirmed cases, 14.1% [95% CI = 0.438, 0.562] were RR (Figure 1).

 The presumptive patients were predominantly males, accounting for 2,930 (63.2%), while female cases constituted 1,707 (36.8%), giving a male/female ratio of 1.71:1. Most patients were adults (45‒54 and 55‒64 years [17.4% and 17.7%, respectively]) and lived in rural areas (55.9%). The related data are provided in Table 1.

**Table 1:** Socio-demographic Characteristics of TB Presumptive Patients in Aceh Referral Hospital, Indonesia, 2015‒2021 (n = 4, 637)

**Variables**	**Number**	**Percent**
Gender		
Male	2930	63.2
Female	1707	36.8
Age (years)		
17‒24	409	8.8
25‒34	578	12.5
35‒44	721	15.5
45‒54	982	21.1
55‒64	1163	25.1
> 65	784	16.9
Residential area		
Urban/Peri-urban	1985	42.8
Rural	2596	55.9
Others	56	1.2
TB results		
Positive	1521	32.8
Negative	3116	67.2

*Note*. TB: Tuberculosis.

###  Frequency of multidrug-resistant tuberculosis among presumptive tuberculosis patients 

**Figure 2:**
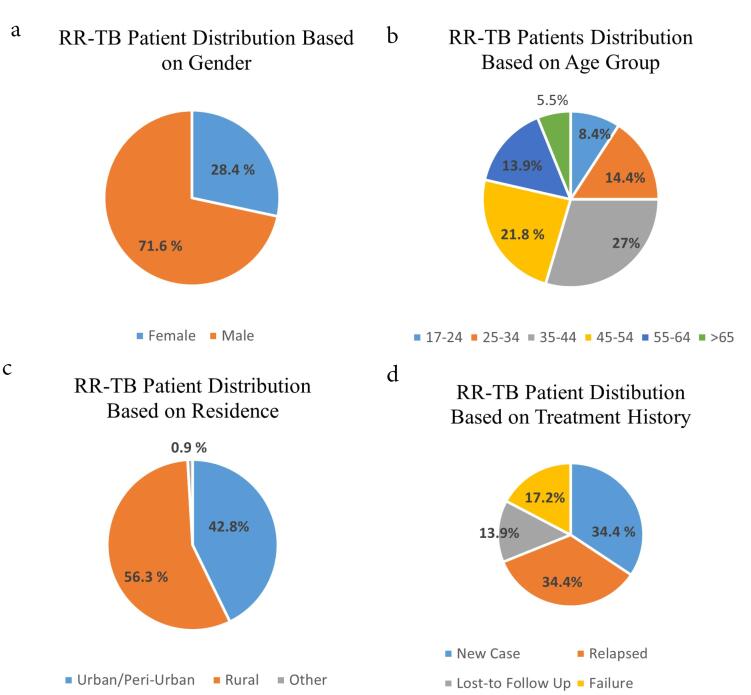
Distribution of RR-TB among presumptive TB patients by (a) gender, (b) age group, (c) patient’s residence, and (d) TB treatment history. *Note*. TB: Tuberculosis

 Of the cases included in the study, 1521 were MTB-detected samples. Among them, RR-TB was found in 215 (14.1%) cases, which comprised 34.4% new TB cases and 65.5% cases having TB treatment before (34.4% relapsed, 13.9% loss-to-follow-up, and 17.2% treatment failure). The proportions of RR-TB by gender, age group, residence, and patient’s treatment history are shown in Figure 2. Most of the patients were males (71.63%), were 35‒54 years old (48.7%), and came from rural areas (56.3%).

###  Frequency of tuberculosis and multidrug-resistant tuberculosis by years

 The findings demonstrated that TB cases increased from 6.04% in 2015 to 23.4% and 21.6% in 2018 and 2019. However, the RR-TB case showed a slight increase from 14.88% in 2015 to 16.74% in 2018 and 19.53% in 2019. A decline in TB case detection occurred in 2020 (14.43%) and 2021 (14.00%). Similarly, the RR-TB cases represented a decline in these 2 years (13.95% and 7.91%, respectively) due to COVID-19 pandemic conditions (Table 2).

**Table 2:** Frequency of RR-TB patients at RSUDZA in 2015‒2021 by years

**Years**	**Presumptive TB patients**		**TB detected**		**RR-TB positive**	
	**Number**	**Percent**	**Number**	**Percent**	**Number**	**Percent**
2015	194	4.09	92	6.04	32	14.88
2016	235	4.95	103	6.77	26	12.09
2017	650	13.70	213	14.01	32	14.88
2018	1,220	25.71	356	23.40	36	16.74
2019	1,141	24.05	329	21.60	42	19.53
2020	700	14.75	215	14.13	30	13.95
2021	605	12.75	213	14.00	17	7.91
Total	4,745	100	1521	100	215	100

*Note*. RR-TB: Rifampicin-resistant tuberculosis.

###  Sociodemographic-associated risk factors of rifampicin-resistant tuberculosis among tuberculosis-positive patients in Aceh referral hospital, Indonesia

 Of the total 1521 TB-confirmed patients, 215 (14.1%) were RR-TB cases. In addition, 35‒44-year-old patients (AOR = 2.11, 95% CI = 1.25, 3.5, *P*< 0.05) were more likely to have RR-TB compared to > 65-year-old patients (Table 3).

**Table 3:** Multivariate logistic regression analysis for the sociodemographic factors associated with RR-TB in Aceh referral hospital, Indonesia, 2015‒2021 (n = 1521)

**Variables**	**Crude OR (95% CI)**	***P*** ** value**	**Adjusted OR (95% CI)**	***P*** ** value**
Gender				
Female	1.01 (0.75, 1.39)	0.913	1.06 (0.78, 1.45)	0.712
Male	1.00		1.00	
Age (y)				
17‒24	1.59 (0.92, 2.70)	0.093	1.63 (0.94, 2.84)	0.084
25‒34	1.65 (0.95, 2.80)	0.074	1.66 (0.95, 2.90)	0.072
35‒44	2.03 (1.20, 3.41)	0.007	2.11 (1.25, 3.50)	0.005
45‒54	1.62 (0.97, 2.71)	0.063	1.65 (0.98, 2.70)	0.067
55‒64	1.15 (0.65, 2.02)	0.603	1.68 (0.66, 2.05)	0.576
> 65	1.00		1.00	
Residential area				
Urban/Peri-urban	1.08 (0.25, 4.81)	0.912	1.05 (0.24, 4.67)	0.954
Rural	1.83 (0.41, 8.10)	0.421	1.79 (0.40, 8.02)	0.435
Other	1.00		1.00	

*Note*. RR-TB: Rifampicin-resistant tuberculosis; OR: Odds ratio; CI: Confidence interval.

## Discussion

 Possessing local data on TB cases and recognizing probable predisposing factors will help arrange a proper intervention method for controlling disease transmission. This study determined the frequency of TB and RR-TB and their associated factors in Aceh Referral Hospital using GeneXpert data collected from January 2015 to December 2021. The positivity of TB among presumptive TB patients in this study was 32.8% (1521/4637). Our frequency result (32.8%) was comparable with previous area reports of TB per 100,000 population in Indonesia, which was 838.7 in Java-Bali, 875 in Sumatra, and 941.2 in other islands.^19^ Differences in diagnostic techniques, population and duration of study, sample size, geographical, and TB control practices are factors that demonstrate the discrepancy of studies.^19-21^

 The presumptive patients’ cases were predominantly males (63.2%), with a ratio of male/female 1.71:1. The difference in the gender proportion of RR-TB might be because men are more exposed to conditions such as overcrowding, low adherence to treatment, smoking habits, and alcohol consumption, making them more vulnerable to RR-TB. It is also in line with the global TB pattern by gender.^22^ A study outlined that young age (10‒25 years) was a potent risk factor for RR-TB cases in Pakistan.^23^ However, a study in Malaysia reported no significant association between the RR-TB and age.^24^ The discordant results revealed no agreement in age and RR-TB because of the difference in age group cut-off points used in studies. Furthermore, the results of the current study are in conformity with reports from the Indonesia Health Ministry, indicating that the highest TB cases were found in productive age groups (25‒34 years old).

 RR-TB cases included 34.4% of new TB cases and 65.5% of cases with TB treatment before. More precisely, patients who had TB drugs before were more likely to suffer RR-TB compared to new cases. A study reported that failure of second-line drugs and primary transmission may have significantly contributed to the number of MDR/RR-TB cases.^13^ The long duration of TB treatment and the risk of drug adverse effects led to patients’ failure to follow up, which contributed to drug resistance. As drug resistance required longer treatment and more complicated adverse effects, studies revealed that not only patients’ good attitudes and practices toward disease were needed but also family support in preventing drug resistance. Continuous educational intervention from medical personnel is also a critical solution to improve TB prevention and treatment success at the family and community levels.

 As rapid identification of MTB and its resistance to rifampicin is important in early disease management, the WHO strongly recommends the Xpert assay for this purpose. It provides not only a rapid diagnostic method as it can detect both MTB and the rifampicin-resistance gene simultaneously but also has better accuracy and negative predictive values than acid-fast bacilli staining. However, GeneXpert is expensive and needs a sophisticated instrument when compared to smear microscopy and culture as the gold standard for TB. Nevertheless, nowadays, Aceh Province has around 47 Xpert machine facilities spread out in the satellite health service facility for active drug-resistant TB patients.^5^ Dr. Zainoel Abidin Hospital was a prior referral hospital in the capital city, serving cases from surrounding regions. The Xpert test was initiated in mid-2014 in this hospital, and the results were reported to the Health Ministry database. This study emphasizes the importance of earlier universal screening of MDR/RR-TB using GeneXpert for patients to have proper treatment immediately.

 The study investigated the dynamic cases of TB and RR-TB in the 7-year study period. Accordingly, it was revealed that the number of TB cases increased from 6.04% in 2015 to 23.4% and 21.6% in 2018 and 2019. However, RR-TB cases showed a slight increase from 14.88% in 2015 to 16.74% in 2018 and 19.53% in 2019. The increase in TB and RR-TB in 2015 may be because GeneXpert in the National TB Program was suggested for patients with TB/HIV co-infection, MDR-TB suspected patients, and children. However, it was then suggested for all presumptive conditions in TB patients. When the total tested number of patients was increased, the positive cases were not significantly different over years. It also conforms to a report in 2019, indicating that the highest number of new TB cases belonged to Southeast Asia (which contributed to 44% of worldwide new cases), with eight countries constituting two-thirds of the global TB incidence, including Indonesia.^3^ This stipulated a prime concern for the TB management program in disease prevention and control in the country.

 A decline in TB case detection occurred in 2020 (14.43%) and 2021 (14.00%). Similarly, the RR-TB cases showed a decline in these 2 years (13.95% and 7.91%, respectively) due to COVID-19 pandemic conditions (Table 2), which profoundly influenced the healthcare sector as the most significant impact. Community physical distancing and efforts to improve COVID-19 treatment facilities by limiting hospital services and converting hospitals to COVID-19 care centers posed a new challenge in infectious disease control programs, including TB elimination. The uncertainty in TB management during the pandemic led to decreased case detection, an increased number of cases, and increased TB mortality rates in the country. The dynamic of the case number in this study also reflects the national prevalence during the study period.^3,25^

 The findings demonstrated the number of TB and RR-TB cases among presumptive TB patients using the GeneXpert MTB/RIF assay and associated sociodemographic factors in Aceh Province Referral Hospital in Indonesia. Nonetheless, the study limitation was related to its retrospective study design using secondary data, which could not explain the causal relationship. Hence, the result of this study should be interpreted with caution.

**Highlights**

- High TB and RR-TB confirmed cases in the Aceh referral hospital.
- GeneXpert MTB/RIF is an important assay for RR-TB rapid diagnosis in endemic areas.
- Previously treated TB patients dominantly contributed to RR/MDR-TB cases.

## Conclusion

 This study reported a major result on the 7-year TB and the number of RR-TB cases and its associated factors in Aceh Referral Hospital, Indonesia. The frequency of TB and RR-TB in this study was 32.8% and 14.1%, respectively. The case detection was found more in males, adults (45‒54 age group), and patients residing in rural areas. The detection rate of RR-TB during the 7-year study period relatively increased before the pandemic and decreased during the pandemic era. Hence, the GeneXpert assay was found to be an important and useful tool to detect RR-TB among presumptive TB patients in the endemic area.

## Acknowledgments

 The authors would like to thank the staff of the clinical microbiology laboratory for their data support.

## Authors’ Contribution

**Conceptualization:** Novi Maulina.

**Data curation:** Azzaura Defadheandra, Novi Maulina.

**Formal analysis:** Ika Waraztuty, Novi Maulina.

**Funding acquisition:** Novi Maulina.

**Investigation:** Novi Maulina, Azzaura Defadheandra.

**Methodology:** Zinatul Hayati.

**Project administration:** Azzaura Defadheandra.

**Resources:** Zinatul Hayati.

**Software:** Zulkarnain Zulkarnain.

**Supervision:** Zinatul Hayati.

**Validation:** Kartini Hasballah.

**Visualization:** Zulkarnain Zulkarnain.

**Writing–original draft:** Novi Maulina.

**Writing–review & editing:** Novi Maulina, Zinatul Hayati.

## Competing Interests

 The authors declare no conflict of interests.

## Ethical Approval

 Ethical clearance was secured from the Institutional Review Board of Dr. Zainoel Abidin Hospital and Faculty of Medicine, Aceh (088/EA/FK-RSUDZA/2022). Permission for data collection was obtained from the Clinical Microbiological Laboratory of the Integrated Clinical Laboratory of Dr. Zainoel Abidin Hospital, Banda Aceh, Indonesia.

## Funding

 This research was supported by the Research Institutions and Community Service (LPPM) of Universitas Syiah Kuala (with contract number 402/UN11.2.1/PT.01.03/PNBP/2023).
